# Catching the Culprit: Benzylpenicillin Neurotoxicity Confirmed by Therapeutic Drug Monitoring in a Critically Ill Patient With Continuous Venovenous Hemofiltration

**DOI:** 10.1097/FTD.0000000000001215

**Published:** 2024-06-06

**Authors:** Thomas G. van Gelder, Valentijn A. Schweitzer, Esther V. Uijtendaal, Maaike A. Sikma

**Affiliations:** * Department of Clinical Pharmacy, University Medical Center Utrecht, Utrecht University, Utrecht, the Netherlands;; † Department of Medical Microbiology, University Medical Center Utrecht, Utrecht University, Utrecht, the Netherlands; and; ‡ Intensive Care and Dutch Poisons Information Center, University Medical Center Utrecht, Utrecht University, Utrecht, the Netherlands.

**Keywords:** benzylpenicillin, neurotoxicity, CRRT, intensive care unit, therapeutic drug monitoring

## Abstract

We present the case of a 65-year-old patient who was treated with high-dose benzylpenicillin for severe invasive pneumococcal pneumonia, complicated by acute renal failure managed with continuous venovenous hemofiltration. After cessation of continuous venovenous hemofiltration, the patient experienced multiple tonic–clonic seizures. Therapeutic drug monitoring revealed high total serum concentrations of benzylpenicillin, identifying it as the likely cause of the neurotoxicity. This case study presents the first documented total serum benzylpenicillin concentration associated with neurotoxicity.

## CLINICIAN

A 65-year-old Caucasian woman, previously only taking statins for hypercholesterolemia, presented to the emergency department with a 1.5-week history of vomiting, diarrhea, speech difficulty, and episodes of fainting. Respiratory deterioration necessitated direct intensive care unit admission and intubation. The patient's clinical course was complicated by acute renal failure, requiring continuous venovenous hemofiltration (CVVH), which was started on day 3. Diagnostic evaluations confirmed severe invasive pneumococcal pneumonia and pleural empyema. By the fifth day, laboratory analysis had identified a strain of *Streptococcus pneumoniae*, susceptible to penicillin (minimum inhibitory concentration 0.016 mg/L). This susceptibility profile prompted the initiation of targeted antibiotic therapy with benzylpenicillin at 7200 mg (12 million IU) continuous intravenous (IV) per day. The clinical scenario became more complex with the subsequent diagnosis of purulent pericarditis, confirmed through pericardiocentesis.

Considering the severity of the disease, the microbiologist suggested increasing the dose of benzylpenicillin to 14,400 mg (24 million IU) continuous IV per day. However, owing to the patient's ongoing CVVH treatment, the dosage was maintained at 7200 mg (12 million IU) continuous IV per day.

CVVH was discontinued on day 35. In the early hours of day 37 (00:30 am), the patient experienced a tonic–clonic seizure characterized by rhythmic jerking movements of her arms and face. At this time, alongside benzylpenicillin (which she had been receiving for the previous 14 days), her IV medications included remifentanil (0.2 mg continuous IV per hour), clonidine (84 μg continuous IV per hour), and furosemide (2 mg continuous IV per hour). Following the seizure, the patient's level of consciousness significantly decreased, with the Richmond Agitation-Sedation Scale score falling to −3 from a baseline of 0. Consequently, the dose of remifentanil was reduced. Shortly thereafter, at approximately 02:00 am, she experienced two more tonic–clonic seizures, entering a postictal phase. Benzylpenicillin infusion was stopped at 05:00 am, and CVVH was restarted at approximately 06:00 am

Could benzylpenicillin be the culprit drug?

## TDM CONSULTANT

Neurotoxicity is a well-known and well-documented complication of high-dose benzylpenicillin, which can lead to serious complications.^1,2^ Delirium, for instance, is one such neurotoxic effect and is associated with increased mortality 0–30 days after hospital discharge.^3,4^

This risk of neurotoxicity is amplified in critically ill patients because of increased drug concentrations in the cerebrospinal fluid (CSF), which can result from systemic inflammation compromising the blood–brain barrier.^5^ In addition, increased toxicity may occur due to increased serum concentrations as a consequence of renal impairment with decreased protein binding.^6^

Did you rule out other possible explanations for the tonic–clonic seizures?

## CLINICIAN

Computed tomography of the brain showed no evidence of intracranial hemorrhage, recent ischemia, or an intracranial abscess. A lumbar puncture conducted at 09:30 pm showed no biochemical signs of meningitis.

Given the severity of our patient's condition, our aim is to achieve optimal pharmacokinetic/pharmacodynamic targets. Specifically, we aim to maintain the drug concentration at 4–5 times the minimum inhibitory concentration for the entire dosing interval (ƒT > minimum inhibitory concentration).^7,8^

This confronts us with a challenging trade-off: balancing the need for aggressive high-dose antibiotic therapy to ensure effective treatment against the risk of increased toxicity. Benzylpenicillin is our preferred treatment, but we need to confirm whether it is the cause of the seizures. If not, we would prefer to continue its use. In the meantime, we have initiated ceftriaxone at a dosage of 4 g continuous IV per day.

Do you have any way of informing us about the patient's exposure to benzylpenicillin?

## TDM CONSULTANT

Therapeutic drug monitoring (TDM) of β-lactam antibiotics has been proposed as a strategy to prevent excessive exposure and resultant neurotoxicity.^9^ This would be especially relevant in critically ill patients undergoing renal replacement therapy, in whom benzylpenicillin clearance is altered.^10^

## CLINICIAN

Are there any reference values for benzylpenicillin available?

## TDM CONSULTANT

Previously reported steady-state total serum concentrations were 13.7 mg/L (range 5.2–53.6 mg/L), achieved through continuous IV administration of 7200 mg or 12 million IU of benzylpenicillin per day.^11^ However, the threshold benzylpenicillin concentration above which neurotoxic events should be anticipated remains unknown.

Could you specify the exact times when the samples were collected?

## CLINICIAN

We drew blood samples at 05:25 am on day 37, 3 hours after the last neurotoxic event and 25 minutes after the discontinuation of benzylpenicillin therapy, and again at 08:20 am, approximately 2 hours after restarting CVVH.

## TDM CONSULTANT

**FIGURE 1.**
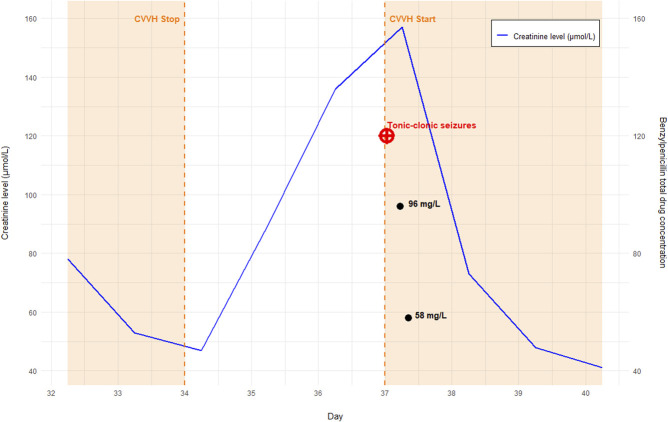
Creatinine level in µmol/L over the course of intensive care unit admission. Within the graph, distinct data points represent tonic–clonic seizure events and total benzylpenicillin drug concentrations. The shaded orange region corresponds to the duration of CVVH.

The results were insightful: we measured a total benzylpenicillin serum concentration of 96 mg/L at 05:25 am and 58 mg/L at 08:20 am. To illustrate the fluctuations in creatinine levels during neurotoxicity, we prepared Figure 1. In addition, a total benzylpenicillin concentration of 7.6 mg/L was detected in the CSF sample collected at 09:30 pm on day 37, confirming the presence of benzylpenicillin in the CSF.

Given these findings, the total serum concentration of 96 mg/L is considered high, suggesting a probable link between the neurotoxic event and benzylpenicillin administration.

## CLINICIAN

This confirmation of benzylpenicillin-induced neurotoxicity leads us to continue treatment with ceftriaxone at a dosage of 4 g per day.

Given the circumstances, do you believe that the 7200 mg (12 million IU) continuous IV per day dosage of benzylpenicillin during CVVH was excessively high?

## TDM CONSULTANT

The challenge of dosing β-lactam antibiotics accurately in patients undergoing continuous renal replacement therapy is widely recognized. Yet, clear dosing recommendations are scarce, with dosing frequently based on empirical judgment.^12^ You were already dosing lower than the microbiologists' recommendation of 14,400 mg (24 million IU), which is not an unusual dose; for instance, it is the recommended dosage for treating neurosyphilis. The chosen maintenance dose of 7200 mg (12 million IU) continuous IV per day during continuous renal replacement therapy is also supported by the literature.^10^ The decision to not lower this daily dose of 7200 mg (12 million IU) even further following the discontinuation of CVVH could be scrutinized, especially in light of several negative cultures. On the other hand, the extensive nature of the infection warranted a cautious approach to reducing the dose.

TDM during CVVH might have supported you by revealing high total benzylpenicillin serum concentrations, which could have led to decreasing the dose before the cessation of CVVH.

## CLINICIAN

Should we consider the integration of TDM for benzylpenicillin and other β-lactam antibiotics into our standard clinical protocols?

## TDM CONSULTANT

The European Society of Intensive Care Medicine and endorsing organizations have recently underscored the importance of TDM for β-lactam antibiotics in critically ill patients, advocating its routine use to balance effective dosing against the likelihood of toxicity.^13^ Research specifically focusing on TDM to characterize β-lactam–associated neurotoxicity has shown that neurotoxicity may still occur despite dose adjustments for renal impairment, thereby reinforcing the value of TDM in providing more detailed and specific information to guide treatment.^14,15^ This case illustrates the complications of high-dose benzylpenicillin therapy despite dose adjustments in a patient with renal failure.

## CONCLUSION

In patients with renal impairment, especially those undergoing renal replacement therapy, monitoring concentrations of benzylpenicillin should be used to optimize therapy and lower the risk of neurotoxicity. We demonstrated that neurotoxicity may occur at a total serum benzylpenicillin concentration of 96 mg/L, offering guidance on the threshold concentration at which benzylpenicillin dosing should be reduced.
